# Therapeutic Applications of Azanucleoside Analogs as DNA Demethylating Agents

**DOI:** 10.3390/epigenomes7030012

**Published:** 2023-07-05

**Authors:** Fallon Gallimore, Tamer E. Fandy

**Affiliations:** Department of Pharmaceutical & Administrative Sciences, School of Pharmacy, University of Charleston, Charleston, WV 25304, USA; fallongallimore@ucwv.edu

**Keywords:** epigenetics, DNA methylation, DNA demethylating agents, leukemia, 5-azanucleosides

## Abstract

Azanucleosides, such as 5-azacytidine and decitabine, are DNA demethylating agents used in the treatment of acute myeloid leukemia and myelodysplastic syndromes. Researchers continue to explore their utility in the treatment of other hematologic and solid tumors. Based on the capacity of the compounds to inhibit DNA methyltransferase enzymes and the important role of DNA methylation in health and disease, it is essential to understand the molecular changes that azanucleosides induce and how these changes may improve treatment outcomes in subsets of patients. This review summarizes the molecular and therapeutic actions of azanucleosides and discusses recent clinical trials of these compounds as single agents or in combination therapy for the treatment of cancer and related conditions.

## 1. Introduction

Epigenetic modifications are the main mechanism underlying normal and abnormal human development. There are numerous modifications found within the human genome at any given time and DNA methylation is among the most common and well-known of these modifications. DNA methylation regulates many important cellular processes, including gene expression and repression, chromatin remodeling, genomic imprinting, and X-chromosome inactivation. It is essential for normal development and aberrant DNA methylation in the form of hypomethylation or hypermethylation can contribute to the development of disease [1]. The azanucleosides 5-azacytidine, decitabine, and guadecitabine interfere with the DNA methylation machinery and demonstrated efficacy in the treatment of myelodysplastic syndromes, blood dyscrasias, and other malignancies resistant to standard chemotherapeutic agents. The focus of this review is mainly on the therapeutic applications of azanucleosides as single agents or in combination therapy and the rationale for these combinations. The multiple mechanisms of antitumor activity of these drugs and their effect on cellular differentiation, cell cycle arrest, and apoptosis are discussed in detail in other reviews [2,3,4].

## 2. DNA Methylation and Demethylation

Histone posttranslational modifications in the mammalian genome include sumoylation, ubiquitylation, acetylation, phosphorylation, citrullination, crotonylation, and methylation. These modifications are regulated by specific enzymes that add or remove the chemical moiety and epigenetically regulate gene expression. Alterations in these epigenetic modifications are associated with aging as well as the development of various cancers and degenerative diseases [1,5]. Among these modifications, DNA methylation and histone acetylation are the most studied, where DNA methylation is involved in the normal development of mammals and key biologic processes, such as X-chromosome inactivation and genomic imprinting [6,7,8,9].

The process of DNA methylation is catalyzed by the DNA methyltransferase (DNMT) family of enzymes. These enzymes facilitate the transfer of a labile methyl group from the methyl donor S-adenosyl-L-methionine (SAM) cofactor to the C5 position of the cytosine ring, forming 5-methylcytosine (5 mC). Mammalian DNMTs include DNMT1, DNMT2, DNMT3A, DNMT3B, and DNMT3L. DNMT1 is a large protein that forms a complex with Ubiquitin-like, containing PHD and RING finger domains 1 (UHRF1) to maintain DNA methylation during the process of DNA replication. DNMT3A and DNMT3B are responsible for establishing methylation patterns during embryonic development. DNMT3L is catalytically inactive but required for gene imprinting and for regulation of activities by DNMT3A and DNMT3B. Lastly, DNMT2 lacks DNMT activity but instead acts as an RNA methyltransferase. In somatic cells, almost all of the DNA methylation occurs in a CpG dinucleotide context. The regions of DNA that contain a large number of CpG dinucleotide repeats are referred to as CpG islands [10].

Methylated DNA is recognized by methyl-CpG-binding domain (MBD) proteins, UHRF proteins, zinc finger-containing proteins, basic leucine zipper-containing transcription factors, and homeodomain-containing transcription factors. MBD-containing proteins, such as MeCP2, MBD1, MBD2, MBD3, and MBD4, are involved in the maintenance and spread of DNA methylation, recruitment of DNMT1 to hemi-methylated DNA, and repression of gene transcription. The MBD domain of MeCP2 is highly specific to methylated CpG sites adjacent to A/T-rich sequences. UHRF proteins, including UHRF1 and UHRF2, maintain DNA methylation by targeting DNMT1 to hemi-methylated DNA during the process of DNA replication. Zinc finger domain proteins, including ZBTB33 (Kaiso), ZBTB4, and ZBTB38, bind to methylated DNA and repress transcription in a DNA methylation-dependent manner [10,11,12].

Adenine bases in the mammalian genome can also undergo SAM-dependent methylation similar to cytosine. Adenine methylation has recently been discovered through the use of deep sequencing technology. This modification is catalyzed by N6-adenine-specific DNMT1 (N6AMT1) and commonly takes place on the amino group at the sixth position of the purine ring. The result is the formation of N6-methyldeoxyadenosine. Demethylation of adenine is accomplished by alkylated DNA repair protein B homolog (ALKBH1). Studies have shown that decreased levels of genomic adenine methylation are associated with tumorigenesis and poor prognosis for cancer patients. Studies have also reported that there is a connection between adenine methylation and mitochondrial DNA damage [10].

**Figure 1:**
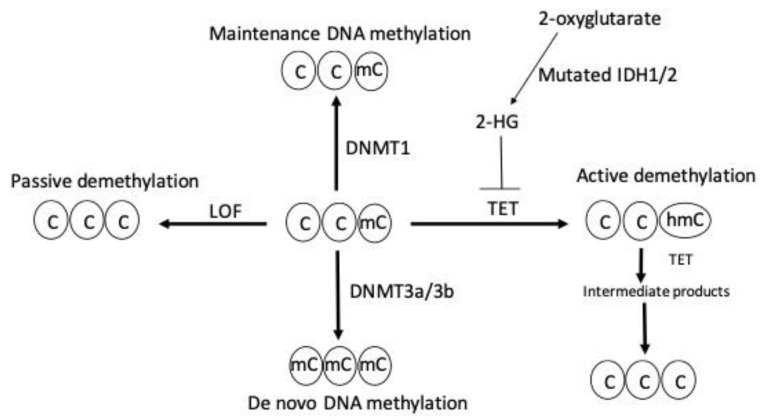
DNA methylation and demethylation. Maintenance and de novo DNA methylation are catalyzed by DNMT1 and DNMT3a/3b, respectively. Loss of function (LOF) mutation of DNMT enzymes induces passive demethylation. TET enzymes catalyze active demethylation by sequential oxidation reactions that leads to the formation of sequential intermediate products, such as 5 hydroxylmethylcytosine (5 hmc), 5-formylcytosine, and, finally, 5-carboxylcytosine, which is replaced by unmodified cytosine through base excision repair. TET enzymes are inhibited by the oncometabolite 2-hydroxyglutarate (2-HG) generated by neomorphic mutations in isocitrate dehydrogenase 1 (IDH1) and IDH2 enzymes resulting in active demethylation inhibition. C indicates cytosine, mC indicates methylcytosine, and hmC indicates 5-hydroxylmethylcytosine.

DNA demethylation is mediated by either an active or passive mechanism. Active DNA demethylation is catalyzed by the ten-eleven tanslocation (TET) proteins. These are Fe(II) and α-ketoglutarate-dependent dioxygenases that utilize molecular oxygen and α-ketoglutarate as co-substrates to generate sequentially oxidized 5 mC derivatives, such as 5-hydroxymethylcytosine, 5-formylcytosine, and 5-carboxycytosine. These entities are recognized and excised by thymine DNA glycosylase, which generates apyrimidinic sites that are subsequently corrected with base excision repair. On the other hand, passive DNA demethylation occurs when 5 mC is lost due to DNMT1 loss of function and successive rounds of DNA replication; this ultimately results in the dilution of the methylated DNA. Figure 1 summarizes the mechanism of DNA methylation and demethylation by passive and active ways.

## 3. Normal and Aberrant DNA Methylation

There are several biological functions of DNA methylation. These include the transcriptional repression of retrotransposons, monoallelic expression of imprinted genes, X-chromosome inactivation in female cells, and regulation of tissue-specific gene expression [11,12,13]. DNA methylation’s repressive effect on gene expression is observed across different regions of the genome. For instance, intergenic regions, including transposable and viral elements, compose nearly 45% of the mammalian genome. DNA methylation plays a repressive role in these regions to avoid their expression and consequent genetic mutations. Likewise, CpG islands in or around promoter regions could be methylated to impair the binding of transcription factors, recruit repressive methyl-binding proteins, and silence gene expression. This is important because the majority of gene promoters, especially for housekeeping genes, reside within CpG islands [12].

Aberrant DNA methylation is characterized by a genome that is generally hypomethylated or hypermethylated. Mechanisms for hypermethylation and hypomethylation include changes in the activity of DNMTs and TET enzymes, structure of chromatin, and selective recruitment of DNA modifying enzymes. These aberrant patterns are known to produce genomic instability and promote pathological conditions, including neurological diseases, immunological diseases, atherosclerosis, osteoporosis, and cancer [14,15,16,17].

Abnormal gene-specific methylation and global hypomethylation contribute to disease in humans. The effects of global DNA hypomethylation are thought to be due to the activation of normally dormant transposable elements within the genome. Activation events could result in changes in transcription factor levels, especially those involved in growth regulation, as well as the accumulation of genomic mutations and abnormal chromosomal recombination. As a result, there is an increase in DNA damage, decondensation of chromatin, and chromosomal instability [16].

Cancers typically display widespread hypomethylation of DNA repetitive elements as well as focal DNA hypermethylation. Hypermethylation of an active CpG-rich promoter often corresponds to repression of the gene’s expression except in cases where there is an alternative unmethylated promoter. Tumor suppressor genes, miRNAs, and other regulatory elements are often targets for this modification in disease states.

## 4. DNA Demethylating Agents

DNA demethylating agents, also known as DNMT inhibitors (DNMTi), are a class of drugs used for a reversal of DNA methylation. There are two main classes of DNA demethylating agents: nucleoside DNMTi and non-nucleoside DNMTi. Nucleoside DNMTi incorporate into DNA, trap DNMTs, facilitate their degradation by cellular proteosomes, and consequently lead to DNA demethylation. Examples of nucleoside DNMTi include 5-azacytidine, decitabine, zebularine, and guadecitabine. On the other hand, the non-nucleoside DNMTi induce DNA demethylation by mechanisms that do require incorporation into DNA. Examples of non-nucleoside DNMTIs include curcumin, procaine, hydralazine, and isooxazoline [18,19].

Azanucleosides (AZN) were first synthesized in 1964, and clinical trials began to demonstrate their anticancer activity shortly after this. The mechanism of action of AZN drugs starts with their cellular uptake, intracellular metabolism, and incorporation into nucleic acids. Incorporation of the 5-aza-dCTP metabolite impairs methylation of DNA by irreversible inhibition of DNMTs. DNMT1 is responsible for the maintenance of methylation patterns during DNA replication; therefore, the inhibition and eventual degradation of this enzyme means that methylation patterns are lost during DNA replication [2,18,19,20].

Azacitidine (5-azacytidine) was the first DNA hypomethylating agent to receive regulatory approval by the FDA in 2004 for the treatment of myelodysplastic syndrome (MDS). Since then, it has also received approval for treatment of acute myeloid leukemia (AML) with 20–30% bone marrow blasts. Azacitidine is mostly incorporated into RNA, and the remaining 10–20% of the drug is incorporated into DNA after a multistep conversion by the enzyme ribonucleotide reductase. The incorporation of azacytidine into DNA and RNA results in DNA damage, growth inhibition, G2 cell cycle arrest, apoptosis, and inhibition of DNA synthesis and repair [18].

Decitabine (5-aza-2′-deoxycytidine) was the second DNA hypomethylating agent to receive regulatory approval by the FDA in 2006 for the treatment of high-risk MDS. Decitabine has a similar chemical structure and mechanism to azacitidine; however, it only incorporates into the DNA, induces apoptosis in a p53-dependent manner, and has greater demethylation potential than azacitidine [19]. Guadecitabine (SGI-110) is a second-generation DNMTi that was designed by chemically linking decitabine to deoxyguanosine by a phosphodiester bond. This design makes the drug less susceptible to degradation by the enzyme cytidine deaminase and is expected to improve the exposure time and degree of marrow penetration by the drug. Early studies with guadecitabine have demonstrated greater DNA hypomethylation activity compared to azacitidine and decitabine [21].

## 5. Therapeutic Applications of AZN

Azacitidine, decitabine, and guadecitabine are primarily used to manage hematologic malignancies, such as MDS and AML [22,23]. The anticancer activity of these drugs is hypothesized to be mediated through inhibiting the activity of DNMTs and consequent reactivation of epigenetically silenced tumor suppressor genes. The reactivation of these genes may lead to various events, including cell differentiation, death, inhibition of proliferation, and sensitization to chemotherapy and immunotherapy. In the tumor microenvironment, AZNs induce an increase in immune responses and decrease in angiogenesis [24,25]. Furthermore, it has been demonstrated that decitabine augments the responsiveness of natural killer cells, which are a part of the body’s innate response against malignant cells [26].

MDS refers to a collection of bone marrow diseases characterized by cytopenia, dysplastic morphology, and genetic evidence of clonality. Cases of MDS are classified based on whether there is single-lineage or multilineage dysplasia, ring sideroblasts, excess blasts, or an isolated del(5q) abnormality [27]. AML is characterized by abnormal proliferation and differentiation of clonal myeloid stem cells. Cases of AML are classified based on whether there are recurrent genetic abnormalities, myelodysplasia-related changes, therapy-related myeloid neoplasms, myeloid sarcomas, and myeloid proliferations related to Down syndrome [28].

### 5.1. Azacitidine

Azacitidine is typically administered in therapeutic cycles as a subcutaneous injection and currently is available orally. Cycles are repeated every four weeks with dosage adjustments made based on hematologic responses and signs of toxicity. If signs of toxicity are observed, it is necessary to delay the start of the next cycle or reduce the dose of the drug. Azacitidine administration is commonly associated with adverse reactions, such as myelosuppression and gastrointestinal toxicities. Studies suggest that azacitidine treatment should be continued for a minimum of six cycles before evaluating the overall impact of the treatment [29].

Early phase I and II trials in the 1970s found azacitidine to be effective in the treatment of myeloid malignancies; most of these studies included patients with relapsed AML [30,31,32,33,34]. Later studies performed by the Cancer and Leukemia Group B demonstrated that azacitidine showed a significant response rate in patients with MDS and AML when given at the current recommended dose of 75 mg/m^2^ daily for seven days [35,36,37,38]. These studies ultimately led to U.S. FDA approval of azacitidine for treatment of myeloid malignancies. Another randomized phase II study, AZA-001, determined the effects of azacitidine on survival. The study revealed that patients treated with azacitidine survived an average of 24.5 months compared to the average 15.0 months survived by patients receiving standard care. Patients treated with azacitidine had significantly greater survival and complete responses as well as lower disease progression, relapse, and death [38,39,40].

A systematic review and meta-analysis revealed that MDS treatment with demethylating agents, specifically azacitidine, prolongs overall survival and time to AML transformation or death. This overall survival advantage was observed despite the higher toxicity profile of azacitidine compared to other AZN. The review examined a total of four trials; two of these trials focused on the effect of azacitidine and included 549 patients. The survival advantage was characterized by a hazard ratio of 0.67 with a 95% confidence interval [0.54, 0.83]. The prolonged time to AML transformation or death compared to conventional care was characterized by a hazard ratio of 0.54 with a 95% confidence interval [0.42, 0.70]. These advantages were not observed with the other two trials focused on the effect of decitabine [41].

### 5.2. Decitabine

Decitabine is typically administered as an intravenous infusion, and there are currently two administration regimens approved by the U.S. FDA. The original six-week regimen consists of continuous intravenous infusion of 15 mg/m^2^ for three hours, which is repeated every eight hours for three days. The four-week regimen consists of continuous intravenous infusion of 20 mg/m^2^ for one hour, which is repeated daily for five days; the reduced infusion time allows for treatment in outpatient settings. Complete blood counts are obtained before each cycle to monitor hematologic responses and signs of toxicity. Like azacitidine, the main toxicities associated with this drug are myelosuppression (i.e., neutropenia and thrombocytopenia) and gastrointestinal toxicities [29].

Early phase II studies demonstrated the selective DNA demethylating activity of decitabine near the current recommended dose. These studies revealed that individuals receiving the original six-week regimen experienced complete responses, partial responses, and hematologic improvement [42,43]. The results from these studies prompted the phase III trial in the U.S. that led to FDA approval of the drug. In this landmark study, decitabine was administered to 170 patients using the six-week regimen, and the patients receiving decitabine had greater overall response rates and hematologic improvement. The decitabine patients also had an average delay of 4.3 months to AML transformation or death, improvements in global health status, and reduction in symptoms such as dyspnea and fatigue [44,45]. Current trials of decitabine have shown some clinical efficacy in treating patients with high-risk MDS with overall response rates ranging from 17% to 32% [44].

Elderly patients with AML is a challenging condition. Most of these patients have poor outcomes after chemotherapy treatment due to the decreased fitness and increased treatment-related mortality associated with this population [46,47,48]. Indeed, advanced age is an adverse prognostic factor in AML patients for several reasons, including comorbid conditions, decreased function of organs, poor performance status, a higher incidence of adverse karyotypes, and increased probabilities of toxicity and fatal side effects from intensive chemotherapy [49,50,51]. An observational study evaluated the efficacy of decitabine in 104 elderly AML patients with a median age of 72.5 years. The overall response rate as a first-line therapy was determined to be 42%, and there was a median overall survival of 12.7 months.

A systematic review and meta-analysis evaluated efficacy and safety outcomes in nine published studies that treated 718 elderly AML patients with decitabine. The efficacy outcomes were defined as complete remission (CR), overall response rate (ORR), and overall survival (OS). The safety outcomes were defined as treatment-related grades 3–4 adverse events and early death rate. Estimates for each outcome were determined using a 95% confidence interval. Estimates for CR, ORR, and OS were 25% (19, 36), 37% (28, 47), and 8.09 months (5.77, 10.41), respectively. Myelosuppression was the most common toxicity observed in these patients. The reported probabilities of common adverse events were a 40% chance of thrombocytopenia (28, 53) and 38% chance of febrile neutropenia (23, 53). Death within 30 days was 7% [2,11] while death within 60 days was determined to be 17% (11, 22). Overall, the data indicate that decitabine is an effective and relatively well-tolerated treatment in elderly AML patients [52].

### 5.3. Guadecitabine

Guadecitabine is a prodrug that has a longer half-life and exposure than its active metabolite decitabine. This is primarily attributed to the drug’s ability to resist immediate degradation by cytidine deaminase. The gradual enzymatic cleavage of guadecitabine results in extended release and prolonged exposure, which will improve the efficacy of AML and MDS treatment with DNA demethylating agents [53,54,55]. A phase I dose-escalation study determined the biologically effective dose for guadecitabine to be 60 mg/m^2^ for five consecutive days in 28-day cycles. This is similar to the dosage schedule that has been approved for decitabine [56,57].

A multicenter, open-label, phase II dose expansion study was conducted to determine the dose-response relation of the 5-day regimen as well as the safety and efficacy of the 10-day regimen. A total of 10 North American academic medical centers participated in this study, and 103 AML patients were randomly assigned to the following groups: 5-day regimen 60 mg/m^2^/d, 5-day regimen 90 mg/m^2^/d, and 10-day regimen 60 mg/m^2^/d. The different doses across the 5-day regimen groups showed no benefit in clinical outcomes. However, composite complete response (CRc) and complete response (CR) rates were generally higher for the 10-day regimen compared to the 5-day regimen (CRc, 30.2% vs. 16.0%, *p* = 0.1061; CR, 18.9% vs. 8%, *p* = 0.15). Adverse events were mainly hematologic in nature and occurred with higher incidence in the 10-day regimen. Early all-cause mortality rates were similar among the different regimens [58].

After azacitdine treatment failure, high-risk MDS and AML patients have very poor survival rates characterized by a median survival of approximately six months. Some studies have shown that guadecitabine has a 52% response rate in treatment naïve AML, and a 32% response rate in patients with relapsed or refractory MDS after initial treatment with DNA demethylating agents [56,57,58,59]. Based on the provided evidence, a multicenter phase II study was conducted to evaluate the efficacy and safety of guadecitabine in high-risk MDS and low blast count AML patients after failed azacitidine treatment; 14.3% patients experienced a complete or partial response, and the median duration of the response was 11.5 months. Patients who responded to the guadecitabine had a median OS of 17.9 months. The above data suggest that guadecitabine may be helpful in treating patients who do not respond to other AZN [60]. However, recent phase III clinical trials in untreated AML patients, previously treated AML patients, and previously treated MDS patients failed to demonstrate a statistically superior survival outcome compared to current therapeutic alternatives.

## 6. AZN Combination Therapies

Combination therapy using AZN has demonstrated the following effects: enhanced radiation sensitivity, increased sensitivity to anticancer drugs, cancer cell reprogramming, and induction of an immune response against cancer. Most of the combinations that have been assessed so far involve the combination of decitabine and platinum-based drugs (e.g., cisplatin and carboplatin). It is important to note that these findings are mostly for the treatment of solid tumors [61]. The rationale behind using combination therapy is to allow for hitting multiple targets and, consequently, sensitize resistant tumors [19].

The antiapoptotic proteins of the B-cell lymphoma 2 (BCL-2) family are commonly overexpressed in leukemic stem cell subpopulations. This suggests that the use of BCL-2 inhibitors in the treatment of AML and MDS will sensitize the tumor cells [62]. Venetoclax (ABT-199) is an oral BCL-2 inhibitor that has currently received FDA approval for the treatment of chronic lymphocytic leukemia (CLL) and small lymphocytic leukemia [19]. These inhibitors, including venetoclax, are known to sensitize the myeloid malignancies to the actions of AZN, such as 5-azacytidine, decitabine, and guadecitabine [63]. Indeed, the combination of 5-azacytidine and venetoclax was demonstrated to block amino acid metabolism, which is crucial to the survival of the leukemic stem cells [64]. This finding ultimately led to the FDA approval of venetoclax in combination with DNMT inhibitors for the treatment of AML patients who cannot undergo intensive chemotherapy.

Histone deacetylases (HDACs) are enzymes that remove the acetyl groups from lysine residues, controlling the remodeling of chromatin, and gene expression. Different classes of HDAC inhibitors, including short chain fatty acids, benzamides, cyclic peptides, hydroxamic acids, and miscellaneous compounds were developed as antitumor agents [65]. A study looking at a murine mammary tumor model used 5-azacytidine in combination with butyrate was found to reduce the number of cancer stem cells and increase overall survival through inhibition of growth-promoting proteins [66].

The combination therapy of DNMTIs and poly ADP-ribose polymerase inhibitors (PARPIs) was also investigated. PARPs are nuclear proteins that are involved in DNA base-excision and single-strand repairs. PARP1 and PARP2 are the main targets of many anticancer drugs due to their specific roles in DNA damage repair [67]. Examples of PARPIs that have shown potential usefulness in the treatment of various cancers include veliparib and talazoparib. A recent study reported that the combination of guadecitabine- and talazoparib-sensitized breast and ovarian cancers are resistant to PARPIs independent of BRCA mutations, which indicates that this combination may be useful in clinical settings [68].

Recently, a combination therapy involving decitabine and cedazuridine was approved by the FDA. The effectiveness of oral decitabine is limited by its extensive metabolism by the enzyme cytidine deaminase. Cedazuridine is responsible for the inhibition of cytidine deaminase in the gastrointestinal tract and liver and, consequently, increases the systemic exposure time of decitabine following oral administration. A fixed oral dose combination of decitabine and cedazuridine is currently being developed for the treatment of MDS, chronic myelomonocytic leukemia (CMML), AML, glioma, and solid tumors. The combination has approval for the treatment of MDS and CMML at this moment. This includes treated and untreated MDS and CMML with specific subtypes (i.e., refractory anemia, refractory anemia with ringed sideroblasts, and refractory anemia with excess blasts) and scores using the International Prognostic Scoring System (i.e., intermediate-1, intermediate-2, and high-risk). The recommended dosage is 35 mg of decitabine and 100 mg of cedazuridine taken for five days on a 28-day cycle with a minimum of four cycles. Furthermore, other studies have shown that the cedazuridine combination therapy increases the bioavailability of decitabine, produces consistent efficacy similar to AZN single agent treatment, and has a tolerability profile similar to IV decitabine [69,70,71].

Resistance development to AZN therapy was correlated with upregulating the expression of PD-1 and PD-L1 in immune T cells. Persistent expression and engagement of PD-1 results in tumor immune evasion. Rational combination of the PD-1 inhibitor nivolumab with AZN showed a higher overall response rate than AZN therapy alone in older AML patients who were ineligible for intensive chemotherapy [72]. Moreover, AZN treatment enhanced responses to anti-CTLA-4 immunotherapy in a melanoma model by activating the endogenous retroviral pathway (ERV). Stimulation of the ERV pathway culminates in T cell activation through IFN production [73].

## 7. AZN in Clinical Trials

Majority of current clinical trials with AZN primarily involve using combination therapy to treat MDS and AML. As discussed above, some combinations have shown promise in treating these conditions and current clinical trials continue to seek out different combinations to enhance the effectiveness of treatment. There are a growing number of clinical trials that investigate the use of AZN in the treatment of other conditions, including other blood and bone marrow disorders, colorectal cancer, lung cancer, breast cancer, prostate cancer, liver cancer, and skin cancer. Table 1 provides a summary of the clinical trials discussed in this section.

### 7.1. Treatment of MDS

A randomized phase II study conducted by Jabbour et al. compared low-dose decitabine and low-dose azacitidine in the treatment of lower-risk MDS and myeloproliferative neoplasm. The aim of the study was to demonstrate the safety and efficacy of AZN treatment for this population. One hundred thirteen patients were randomly assigned to the treatment groups—35% of patients received azacitidine, and 65% received decitabine. Of these patients, 81% were characterized as intermediate 1-risk patients. The overall response rates (ORRs) for azacitidine and decitabine were 49% and 70% (*p* = 0.03), respectively. More patients treated with decitabine became transfusion independent compared to the azacitidine patients (32% vs. 16%, *p* = 0.2). The cytogenetic response rate for decitabine was also greater than the rate for azacitidine (61% vs. 25%, *p* = 0.02). Overall, the treatment was well-tolerated, which is demonstrated by the six-week mortality rate of 0% [74].

A phase 1b/II dose escalation study conducted by Sallman et al. investigated the use of eprenetapopt (APR-246) and azactidine for the treatment of TP53-mutant MDS. The percentage of patients with this condition that achieve CR with AZN is relatively low. Therefore, APR-246 was utilized as it is known to restore wild-type p53 functions in the mutant cells. The sample size of the study was 55 patients—40 patients with MDS, 11 patients with AML, and four patients with MDS/myeloproliferative neoplasms. Each patient that was treated in the study had at least one TP53 mutation. For all groups, the ORR was 71%, and 44% of patients achieved CR. Of the MDS patients, 73% responded to treatment, 50% achieved CR, and 58% experienced a cytogenetic response. Of the AML patients, 64% responded to treatment and 36% achieved CR. Patients responding to the treatment also had significant reductions in TP53 variant allele frequency, and the median overall survival was significantly improved compared to non-responding patients [75].

### 7.2. Treatment of Colorectal Cancer

A phase II single-arm trial conducted by Kuang et al. [76] investigated the use of pembrolizumab and 5-azacytidine in patients with refractory metastatic colorectal cancer. Pembrolizumab inhibits the programmed death-1 (PD-1) receptor, an integral component of immune checkpoint regulation in the tumor microenvironment. The sample size of the study was 30 chemotherapy-refractory patients. The ORR was 3%, median progression-free survival (PFS) was 1.9 months, and median overall survival (OS) was 6.3 months. One patient achieved a partial response, and one patient was determined to have a stable disease. The clinical activity of this combination therapy was limited, so the study did not achieve its goal of treating a total of 40 patients. However, the combination regimen was well-tolerated by these patients, as majority of treatment-related adverse events were grades 1–2. An analysis of biomarkers revealed that the majority of patients receiving the treatment had significantly decreased DNA methylation in gene promoter regions [76].

### 7.3. Treatment of Ovarian Cancer

A randomized phase II trial conducted by Oza et al., used guadecitabine and carboplatin as a treatment method for platinum-resistant, recurrent ovarian cancer. In the study, 100 patients were treated with either the guadecitabine–carboplatin combination or a treatment of choice (i.e., topotecan, pegylated liposomal doxorubicin, paclitaxel, or gemcitabine) in 28-day cycles until progression or significant toxicity was observed. The median PFS was not statistically different (16.3 weeks vs. 9.1 weeks, *p* = 0.07) between the groups. The OS rate, ORR, and clinical benefit response were also not significantly different between groups. However, the six-month PFS rate was significantly higher in the combination therapy group compared to the group receiving treatment of choice (37% vs. 11%, *p* = 0.003). The combination therapy may be enhanced by identifying predictive markers for patient selection [77].

### 7.4. Treatment of Melanoma

A phase I/II trial conducted by Tawbi et al. investigated the safety and efficacy of a decitabine and temozolomide (TMZ) combination therapy for patients with metastatic melanoma. TMZ is commonly used for chemotherapy of metastatic melanoma and combination with decitabine may sensitize resistant melanoma cells to its cytotoxic effect. The phase II portion of the study treated a total of 35 patients. Of these 35 patients, there were 2 complete responses, 4 partial responses, 14 stable disease, and 13 progressive disease. The ORR and clinical benefit rate were determined to be 18%. The median OS was 12.4 months, and the one-year OS rate was 56%. Regarding toxicity and adverse events, grade 3/4 neutropenia, grade 1/2 fatigue, and grade 1 nausea were common among the patients. The results indicate that this combination therapy is relatively safe and needs to be further investigated in a randomized setting [78].

### 7.5. Treatment of Other Blood Dyscrasias

A pilot study conducted by Olivieri et al. investigated the efficacy of decitabine in the treatment of β-thalassemia intermedia (TI). This genetic condition is characterized by moderate-to-severe anemia as a result of ineffective erythropoiesis (i.e., premature red blood cell death). Patients with TI may have a shortened life span, progressive organ damage, and a lower quality of life due to the nature of this disease. A milder course of TI occurs in patients with higher levels of fetal hemoglobin (HbF), which is associated with more effective erythropoiesis and a better prognosis. Researchers hypothesized that decitabine may be effective at increasing hemoglobin (Hb) levels in TI patients by depleting DNMT1 and activating the β-globin gene, which is supposed to correct the imbalance between α and β chains.

Six patients were enrolled in the study, but one patient withdrew after the second week due to fatigue requiring transfusion. The primary outcome, which was an increase in total Hb of 1.5 g/dL or more from baseline to peak, was achieved in two of the patients. For the entire group, Hb increased from baseline 7.88 ± 0.88 g/dL to peak 9.04 ± 0.77 g/dL (*p* = 0.004). Absolute HbF also increased from baseline 3.64 ± 1.13 g/dL to peak 4.29 ± 1.13 g/dL (*p* = 0.003). The trends for increasing Hb and HbF were observed in all five patients that received decitabine treatment. In addition to this, researchers observed a significant decrease in the absolute reticulocyte count (*p* = 0.039) and a significant shift toward normalization of red blood cell Hb concentration (*p* = 0.022). The results of the study indicate that decitabine can be used in the treatment of TI without major cytotoxic effects, but this requires further investigation and clinical evaluation [79].

An open-label, three arm study conducted by Tang et al., investigated the use of low-dose decitabine for refractory prolonged isolated thrombocytopenia (RPIT) after an allogenic hematopoietic cell transplantation (HCT). A total of 97 patients were enrolled in the study and randomly assigned to three arms: low-dose decitabine and recombinant human thrombopoietin (Arm A), decitabine alone (Arm B), and conventional treatment (Arm C). By the end of the study, only 91 patients were able to be evaluated for primary and secondary end points. Response rates for the three arms were 66.7%, 73.3%, and 19.4%, respectively. One-year survival rates for arms A (64.4 ± 9.1%) and B (73.4 ± 8.8%) were similar, but both were superior to the rate for arm C (41.0 ± 9.8%, *p* = 0.025). In patients that responded to decitabine, the megakaryocytes, endothelial cells, and cytokines involved in megakaryocyte migration and cell damage were improved. The study suggests that low-dose decitabine improves platelet recovery and overall survival in RPIT patients after HCT [80].

## 8. Conclusions

Despite the mainstay role of chemotherapy in the treatment of AML and other types of hematologic malignancies, the ineligibility of some patients for chemotherapy is a major limitation. Epigenetic therapy represented by HDAC inhibitors, DNA demethylating agents, and other novel therapies may be promising alternatives for unfit patients with further optimization of dosing and combination therapies. Current research and clinical trials have demonstrated the clinical efficacy of AZN in treating various cancers and bone marrow disorders. However, drawbacks like the inability to predict a response to therapy and the lack of biomarkers that could predict clinical response are hurdles that limit their use. Surprisingly, DNA methylation reversal cannot be used as a marker to predict the response to AZN, which indicates the importance of other mechanisms in mediating clinical responses. Moreover, the combination therapy of AZN with other epigenetic modifiers, such as HDAC inhibitors, is still empiric due to the lack of understanding of how these drugs interact together to induce a synergistic effect. Resistance development is another therapeutic limitation of AZN with an approximate 50% response rate. The mechanisms of resistance to AZN are not clear but may involve drug transporters and metabolic enzymes relevant to the uptake and metabolism of AZN, respectively.

## Figures and Tables

**Table 1:** Clinical trials of AZNs in both hematologic malignancies and solid tumors.

Study	Identifier	Study Population	Intervention(s)	Primary Outcome Measure(s)	Primary Outcome Measure Results
Jabbour et al., 2017 [74]	NCT1720225	IPSS low- or intermediate 1-risk MDS or CMML	Decitabine 20 mg/m^2^ IV 1 h daily for three days OR azacitidine 75 mg/m^2^ IV 1 h daily or subcutaneously for 3 days (repeated every 4 weeks)	Overall response rate (ORR)	Decitabine ORR: 70% Azacitidine ORR: 49% (*p* = 0.03)
Sallman et al., 2021 [75]	NCT03745716	IPSS low- or intermediate 1-risk MDS, at least one TP53 mutation and isolated deletion of 5q, failure of prior treatment with at least 4 full cycles of lenalidomide	Dose escalation of APR-246 via IV starting at 50 mg/kg lean body weight; azacitidine administered subcutaneously or via IV at 75 mg/m^2^	Complete response (CR) rate	CR for MDS patients:50% CR for AML patients:36%
Kuang et al., 2022 [76]	NCT02260440	Histologically confirmed metastatic colorectal cancer previously treated with currently approved standard therapies	Pembrolizumab 200 mg every 21 days; azacitidine 100 mg subcutaneous injection on days 1–5 every 21 days	Overall response rate (ORR)	ORR: 3%
Oza et al., 2020 [77]	NCT01696032	Platinum-resistant histologically or cytologically confirmed recurrent ovarian cancer; high-grade serous or grade 2–3 endometroid/mixed cell/clear cell epithelial ovarian cancer; primary peritoneal carcinoma; fallopian tube cancer	Guadecitabine 30 mg/m^2^ subcutaneously once-daily on days 1–5 and carboplatin IV AUC 4 on Day 8 OR treatment of choice	Progression free survival (PFS)	Median PFS: 16.3 vs. 9.1 weeks, *p* = 0.076-month PFS rate: 37% vs. 11%, *p* = 0.003
Tawbi et al., 2013 [78]	NCT00715793	Non-resectable stage IIIB/C or stage IV metastatic melanoma; either no prior therapy or have progressed despite prior therapies	Decitabine 0.075 or 0.15 mg/kg IV daily for 5 days/week for 2 weeks; TMZ orally 75 mg/m^2^ for weeks 2–5 of a 6-week cycle	Overall response rate (ORR)	ORR: 18%
Olivieri et al., 2011 [79]	NCT00661726	Beta-thalassemia as confirmed by DNA testing; transfusion independent for at least 120 days; red blood cell folate levels above lower limit of normal	Decitabine 0.2 mg/kg subcutaneously twice a week for 12 weeks	Number of patients with increase in Hb at least 1.5 g/dL and change in total hemoglobin from baseline to peak	# of patients: 2/5 Change in Hb: 7.88 +/−0.88 g/dL (baseline) to 9.04 +/−0.77 g/dL (peak)
Tang et al., 2021 [80]	NCT02487563	Platelet count ≤ 30 × 109/L persistently at day 60 post-HSCT or later; neutrophil and hemoglobin were well recovered; full donor chimerism was achieved; no response to conventional treatments for a duration of at least 4 weeks;	Low-dose decitabine (15 mg/m^2^ daily IV for 3 consecutive days [days 1–3]) plus recombinant human thrombopoietin (300 U/kg daily); decitabine alone; or conventional treatment	Response rate of platelet recovery	Combination: 66.7% Decitabine alone: 73.3% Conventional: 19.4% (*p* < 0.001)
